# When Rare Meets Routine: Anesthetic Management of a Child With Gould Syndrome—A Case Report

**DOI:** 10.1155/crpe/2066149

**Published:** 2026-10-12

**Authors:** Hannah Anderson, Abdul Waleed, Andrew Purcell

**Affiliations:** ^1^ Department of Internal Medicine Santa Barbara Cottage Hospital Santa Barbara California USA cottagehealth.org; ^2^ Department of Anesthesiology and Pain Medicine South Infirmary Victoria University Hospital Cork County Cork Ireland sivuh.ie

**Keywords:** anesthetic management, general anesthesia, pediatric anesthesia, perioperative care, rare genetic disorders

## Abstract

Gould syndrome is a rare, inherited syndrome. Its clinical manifestation may necessitate surgical treatment and anesthetic care. We report a case of anesthetic management of elective, bilateral adductor and gracilis tendon release in a child with a diagnosis of Gould syndrome. As there are currently no reports in the medical literature addressing anesthetic conduct for this condition, we discuss the perioperative considerations necessary to anticipate and mitigate potential complications. The syndrome’s diverse presentation, ranging from neurological deficits and seizures to ophthalmic complications and supraventricular arrhythmias, demands a tailored anesthetic approach.

## 1. Introduction

Gould Syndrome, first identified in 2003, is caused by variants in the *COL4A1* or *COL4A2* genes, usually a missense substitution, which encode the alpha‐1 and alpha‐2 subunits of collagen type IV—a major component of basement membranes [1]. Hereditary transmission typically follows an autosomal dominant inheritance pattern, however, observed phenotypic variation is due to incomplete penetrance and variable expressivity caused by allelic heterogeneity and variable tissue impact [2]. These variants can also occur do novo, and are thought to be more severe than the hereditary variants [3].

The clinical manifestations of *COL4A1* variants in humans represent only a subset of the phenotypes observed in animal models [1]. Neurologically, *COL4A1*‐related disorders can present with a wide range of symptoms, including infantile hemiplegia, developmental delay, intellectual disability, seizures, and intracerebral hemorrhagic and ischemic insults which can occur at any stage of life. Ophthalmic symptoms such as transient visual loss, retinal hemorrhage, cataracts, glaucoma, and microphthalmia or anophthalmia are also common. Additional systemic findings may include nephropathy, Raynaud phenomenon, supraventricular arrhythmias, muscle cramps and even rhabdomyolysis [4–6].

Disorders related to *COL4A1* and *COL4A2* variants are likely underdiagnosed due to their variable expressivity. Pathogenic variants are estimated to contribute to approximately 20% of fetal intracerebral hemorrhage cases and 16% of porencephaly cases [7]. To date, over 400 families with a pathogenic variant have been identified in the literature, presenting with a broad symptomatic range including migraines, hemiparesis, seizures, dystonia, and hydrocephalus. The spectrum of cerebrovascular disease associated with these variants spans from milder forms of cerebral small vessel disease to porencephaly, which typically manifests as fluid‐filled cysts or cavities in the brain resulting from pre‐ or perinatal ischemic and hemorrhagic injuries [7]. Frequency of ischemic and hemorrhagic complications can differ with variant type, with hemorrhagic insults seemingly showing higher frequency in *COL4A2* variant compared to *COL4A1*. Similarly, ischemic event frequency is reported to be higher in *COL4A1* variants [8]. Due to the manifestations of the disease, a proportion of patients with Gould Syndrome will need surgery and anesthesia management, which can be a critical event with increased risk of bleeding or ischemia.

We present the case of a 12‐year‐old boy with a diagnosis of Gould syndrome who underwent general anesthesia for elective bilateral adductor and gracilis tendon release due to chronic spasticity (a common feature of porencephaly) and associated hip displacement. At the time of writing there were no reports detailing the provision of anesthesia in patients with Gould syndrome identifiable in the clinical literature.

## 2. Case Presentation

The patient was diagnosed with sporadic Gould syndrome at 11 years of age following genetic testing, available patient records did not specify which variant was present. Formal diagnosis came after many years of investigations for other medical issues that could be attributed to his condition. Prior to his diagnosis he had experienced focal seizures with postictal Todd’s paresis. Multiple MRIs of his brain (the most recent of which was six months prior to the described surgery) have demonstrated stable, increased signal on T2/FLAIR extending from the periventricular region into the subcortical white matter of the frontal, parietal and occipital lobes. Other changes include stable thinning of the corpus callosum with peri‐ventricular cyst formation adjacent to the frontal horns and bodies of the lateral ventricles; his imaging changes have been consistently reported as stable peri‐ventricular leukomalacia. CT angiography two years prior to this surgery failed to demonstrate any evidence of large vessel occlusion, aneurysmal disease, dissection or arteriovenous malformation. Given the absence of any historical or chronic changes associated with cerebral hemorrhage and the extent of leukomalacia on pre‐existing imaging, it is possible that the variant in question was *COL4A1*. Until now, he has suffered from varying degrees of asymmetric spastic quadriparesis. He mobilizes with a wheelchair and uses a Dynamic Movement Orthoses (DMO) suit, which aids stabilization and proprioception. He undergoes regular interval renal ultrasounds having been diagnosed with a bladder elongation following work‐up for an episode of hematuria. Visual problems include an upward gaze palsy, and diplopia for which he wears glasses to correct. He displays no evidence of cognitive or intellectual disability.

Given the paucity of information in the literature on anesthetic management in this condition, our plan revolved around mitigating any potential anesthetic risks that could have presented given the clinical manifestations of Gould syndrome in general and the patient’s specific needs.

Although our patient had previously had inhalational induction of anesthesia for other surgeries, we opted for an intravenous induction in this instance. The patient had expressed a preference for induction in this manner given some anxiety relating to facemask equipment for inhalational inductions. A eutectic mixture of local anesthesia cream (EMLA) was applied to potential sites of intravenous cannula insertion and a pre‐medication of oral midazolam at a dose of 0.5 mg/kg was administered 30 min prior to transfer to the operating theatre.

Standard monitoring as per Association of Anaesthestists Guidelines were applied [9]. A non‐invasive blood pressure (NIBP) monitoring cuff was applied to the right upper arm with measurements obtained at three‐minute intervals. A 22G intravenous cannula was inserted in the dorsum of the left hand. Pre‐medication with midazolam achieved the desired level of anxiolysis and the patient tolerated pre‐oxygenation without issue. Induction of anesthesia was commenced with fentanyl (1 μg/kg) and propofol (4 mg/kg). Although there is no known contra‐indication to non‐depolarising neuromuscular blockade, as a precautionary clinical decision, we chose to avoid its use for patient comfort in the post‐operative phase given the patients history of muscle cramps at baseline.

We utilised McGrath video laryngoscope to facilitate tracheal intubation with a size 5.5 mm cuffed tracheal tube. A mixture of sevoflurane and nitrous oxide with an FiO2 of 0.4 was used for maintenance of anaesthesia with an age adjusted target minimum alveolar concentration (MAC) of 1.0. Nitrous oxide was utilised to decrease sevoflurane requirements to minimise its potential hemodynamic effects. A multimodal analgesic regimen was utilised intravenously including paracetamol (15 mg/kg), fentanyl (1 μg/kg), ketamine (0.25 mg/kg) and clonidine (0.5 μg/kg) with surgical site infiltration with bupivacaine 0.5% w/v with epinephrine 1:200,000. We opted against using NSAIDs given the patient’s history of hematuria. Fluid management involved administration of 250 mL balanced crystalloid solution (Hartmann’s). A standard anti‐emetic cocktail of dexamethasone and ondansetron were administered also. Mean arterial pressure (MAP) was maintained in a range between 63 and 84 mmHg with the sinus rhythm observed throughout. Surgery proceeded uneventfully and emergence and extubation were unremarkable. The entire case took one hour and 50 min.

Given the patient’s susceptibility to renal complications, particular care was taken to ensure that peri‐operative analgesia remained non‐nephrotoxic, continuing the multimodal approach in the recovery phase. The patient was comfortable post‐operatively, without any post‐intervention cognitive concerns, so we continued with an analgesic regimen including regular paracetamol 15 mg/kg orally with as needed clonidine 1 mcg/kg orally twice daily. The patient was at baseline overnight without any additional analgesic needs and was discharged home the following morning.

The only notable issue post‐operatively was the presence of a non‐blanching rash in the right proximal arm. This area corresponded to the site of the NIBP cuff. The rash featured linear petechiae possibly suggestive of acute rupture of the dermal capillaries. Interestingly the parents noted that petechiae at the NIBP site occurred during previous general anesthetics however it resolved spontaneously within a number of weeks post operatively. As it had not previously caused the patient any pain or distress, the parents were unconcerned by it. In the absence of any other evidence of bleeding/bruising that may have been suggestive of thrombocytopenia/coagulation dysfunction, a clinical decision was made not to subject the patient to unnecessary phlebotomy as ultimately no further management beyond observation was deemed necessary.

## 3. Discussion

Gould syndrome, a rare genetic disorder, can present unique challenges in the perioperative period. There is no identifiable information in the medical literature regarding anesthetic management in cases of Gould syndrome. While minimising surgical stress response is a universal goal in the provision of general anesthesia for surgical procedures, in this case it was important to avoid hemodynamic instability in the face of the potentially increased risk of intracerebral ischaemia/haemorrhage. Similarly, care should be given to minimise exposure to potentially nephrotoxic agents (e.g. NSAIDs); this can present a challenge in peri‐operative pain management. There are reports of rhabdomyolysis in those with both *COL4A1* and *COL4A2* variants [4, 5]. With that in mind, particular attention should also be paid to pressure point protection. Similarly, consideration needs to be given to administration of any agent associated with increased risks of rhabdomyolysis (e.g. suxamethonium). Given that both volatile anesthetic agents and prolonged propofol infusions can be associated with increased risk of rhabdomyolysis, maintenance agent of choice needs to take into consideration projected duration of surgery. As our case was short it was felt sevoflurane use was low risk in this context.

Despite our best efforts the NIBP cuff was likely responsible for the described petechial rash. A number of mechanisms could possibly explain this phenomenon. It is possible that microvascular fragility in the face of repeated NIBP measurements during the surgery may have played a role. *COL4A* variants can lead to significant endothelial dysfunction and disturbed vascular tone. Microvascular rupture secondary to NIBP measurement has been described separately elsewhere. The Rumpel‐Leede sign refers to the acute development of a petechial rash where a cuff has been typically applied to the upper arm; it is described as being caused by acute rupture of the dermal capillaries, signifying microvascular fragility [10]. It should be noted that in this case, other mechanisms not necessarily related to pathological microvascular fragility could possibly account for the petechial rash observed. As we did not have a contemporaneous platelet count, it is not possible to discount potential thrombocytopenia as a possible cause. Another explanation could be as simple as low mechanical resilience in a child, although we feel this would be somewhat coincidental in this instance given the fact that we have not observed or witnessed such a clinical phenomenon in the course of providing anesthesia to thousands of other patients thus far in our careers.

While standard NIBP monitoring is considered entirely reasonable for short surgeries in these cases, consideration should be given to periodic inspection of the NIBP site and potentially intermittently altering the site of measurement during the surgery to mitigate against any potential microvascular damage. Although our patient only exhibited signs of superficial capillary rupture, there is a latent risk of sudden hemodynamic instability or vessel wall failure in these patients.

Patients with Gould syndrome can present with a spectrum of difficult clinical presentations. In cases where Gould syndrome has been diagnosed but there is no prior anesthetic history, careful consideration should be given to pre‐operative assessment and the potential clinical features that could warrant more focused attention. Knowledge of variant type can potentially help identify case‐specific vulnerability and safety concerns. This was unfortunately not identifiable in our case from the patient’s parents or the medical records available to us. We acknowledge that as a single case report there are limits to any recommendations that can be made on the back of our observations. Future case series on this syndrome would better help guide anesthetic management more specifically. Given the paucity of information published in this regard, the patient’s parents were highly supportive of our efforts to document and present our findings and considerations for the benefit of other patients with a Gould syndrome diagnosis who may require general anesthesia.

## Funding

The authors have received no funding with respect to this publication.

## Ethics Statement

Written informed consent was obtained from the patient’s parents. No identifying patient information has been included in this manuscript. Institutional Review Board approval was not required.

## Conflicts of Interest

The authors have no conflicts of interest to declare.

## Supporting Information

Additional supporting information can be found online in the Supporting Information section.

## Supporting information


**Supporting Information** CARE‐checklist_Aug26.

## Data Availability

The data that support the findings of this study are available on request from the corresponding author. The data are not publicly available due to privacy or ethical restrictions.

## References

[1] Kuo D. S. , Labelle-Dumais C. , and Gould D. B. , COL4A1 and COL4A2 Mutations and Disease: Insights Into Pathogenic Mechanisms and Potential Therapeutic Targets, Human Molecular Genetics. (2012) 21, no. R1, R97–R110, 10.1093/hmg/dds346. PMC3459649 22914737

[2] Meuwissen M. E. C. , Halley D. J. J. , Smit L. S. et al., The Expanding Phenotype of Col4A1 and Col4A2 Mutations: Clinical Data on 13 Newly Identified Families and a Review of the Literature, Genetics in Medicine. (2015) 17, no. 11, 843–853, 10.1038/gim.2014.210. 25719457

[3] Tambala D. , Vassar R. , and Musolino P. L. , COL4A1 and COL4A2-Related Disorders: Clinical Features, Diagnostic Guidelines, and Management, Genetics in Medicine. (2025) 27, no. 9, 10.1016/j.gim.2025.101514. 40616396

[4] Plaisier E. and Ronco P. , Adam M. P. , Feldman J. , Mirzaa G. M. et al., COL4A1-Related Disorders, Genereviews, 2009, University of Washington, Seattle, Seattle (WA), 1993–2024, https://www.ncbi.nlm.nih.gov/books/NBK7046/. 20301768

[5] Satomi Okano S. , Shimada S. , Tanaka R. et al., Life-Threatening Muscle Complications of COL4A1-Related Disorder, Brain and Development. (2020) 42, no. 1, 93–97, 10.1016/j.braindev.2019.09.001. 31540749

[6] Olarewaju B. A. , Tejon J. , Shurrab S. et al., COL4A2-Related Disorder Presenting in Adulthood With Rhabdomyolysis, American Journal of Medical Genetics. (2025) 197, no. 4, 10.1002/ajmg.a.63965. 39679724

[7] National Organization for Rare Disorders (Nord) , Rare Diseases: Col4A1/A2-Related Disorders, 2023, https://rarediseases.org/rare-diseases/col4a1-a2-related-disorders/.

[8] Whitaker et al., Systematic Review of Cerebral Phenotypes Associated With Monogenic Cerebral Small‐Vessel Disease, Journal of the American Heart Association. (2022) 11, no. 12, 10.1161/JAHA.121.025629. PMC9238640 35699195

[9] Association of Anaesthetists , Recommendations for Standards of Monitoring During Anaesthesia and Recovery, 2021, https://anaesthetists.org/Portals/0/PDFs/Guidelines%20PDFs/Recommendations%20for%20standards%20of%20monitoring%20during%20anaesthesia%20and%20recovery%202021.pdf?ver=2021-05-26-141701-007.

[10] van Rhijn B. D. , Vlachojannis G. J. , and Balak D. M. W. , Petechial Purpuric Rash After Non-Invasive Blood Pressure Measurement: Rumpel-Leede Sign, BMJ Case Reports. (2019) 12, no. 10, 10.1136/bcr-2019-231541. PMC6803111 31604722

