# Supplementary material for: When Rare Meets Routine: Anesthetic Management of a Child With Gould Syndrome—A Case Report
Source: Case Rep Pediatr. 2026 Oct 12;2026:2066149. doi: 10.1155/crpe/2066149 (PMC13659058; doi:10.1155/crpe/2066149)
Supplement: Supplementary file 1 [file CRPE-2026-2066149-s001.pdf]

CARE Checklist of information to include when writing a case report

CARE case report guidelines

(cc) BY-NC-ND

Topic	Item	Checklist item description	Reported on Line
Title	1	The diagnosis or intervention of primary focus followed by the words "case report"	1
Key Words	2	2 to 5 key words that identify diagnoses or interventions in this case report, including "case report"	14
Abstract (no references)	3a	Introduction: What is unique about this case and what does it add to the scientific literature?	17
	3b	Main symptoms and/or important clinical findings	19
	3c	The main diagnoses, therapeutic interventions, and outcomes	21
	3d	Conclusion—What is the main "take-away" lesson(s) from this case?	23
Introduction	4	One or two paragraphs summarizing why this case is unique (**may include references**)	28
Patient Information	5a	De-identified patient specific information.	58
	5b	Primary concerns and symptoms of the patient.	74
	5c	Medical, family, and psycho-social history including relevant genetic information	65
	5d	Relevant past interventions with outcomes	84
Clinical Findings	6	Describe significant physical examination (PE) and important clinical findings.	77
Timeline	7	Historical and current information from this episode of care organized as a timeline	65
Diagnostic Assessment	8a	Diagnostic testing (such as PE, laboratory testing, imaging, surveys).	69
	8b	Diagnostic challenges (such as access to testing, financial, or cultural)	169
	8c	Diagnosis (including other diagnoses considered)	N/A
	8d	Prognosis (such as staging in oncology) where applicable	N/A
Therapeutic Intervention	9a	Types of therapeutic intervention (such as pharmacologic, surgical, preventive, self-care)	96
	9b	Administration of therapeutic intervention (such as dosage, strength, duration)	96
	9c	Changes in therapeutic intervention (with rationale)	98
Follow-up and Outcomes	10a	Clinician and patient-assessed outcomes (if available)	122
	10b	Important follow-up diagnostic and other test results	130
	10c	Intervention adherence and tolerability (How was this assessed?)	121
	10d	Adverse and unanticipated events	124
Discussion	11a	A scientific discussion of the strengths AND limitations associated with this case report	166
	11b	Discussion of the relevant medical literature **with references**.	133
	11c	The scientific rationale for any conclusions (including assessment of possible causes)	153
	11d	The primary "take-away" lessons of this case report (without references) in a one paragraph conclusion	161
Patient Perspective	12	The patient should share their perspective in one to two paragraphs on the treatment(s) they received	N/A
Informed Consent	13	Did the patient give informed consent? Please provide if requested	Yes ☒ No ☐
